# Synergistic Effect of MiR-146a Mimic and Cetuximab on Hepatocellular Carcinoma Cells

**DOI:** 10.1155/2014/384121

**Published:** 2014-05-07

**Authors:** Suning Huang, Rongquan He, Minhua Rong, Yiwu Dang, Gang Chen

**Affiliations:** ^1^Department of Radiotherapy, First Affiliated Hospital of Guangxi Medical University, 6 Shuangyong Road, Nanning, Guangxi Zhuang Autonomous Region 530021, China; ^2^Department of Medical Oncology, First Affiliated Hospital of Guangxi Medical University, 6 Shuangyong Road, Nanning, Guangxi Zhuang Autonomous Region 530021, China; ^3^Research Department, Affiliated Cancer Hospital, Guangxi Medical University, 71 Hedi Road, Nanning, Guangxi Zhuang Autonomous Region 530021, China; ^4^Department of Pathology, First Affiliated Hospital of Guangxi Medical University, 6 Shuangyong Road, Nanning, Guangxi Zhuang Autonomous Region 530021, China

## Abstract

Previously, we found that the expression of microRNA-146a (miR-146a) was downregulated in hepatocellular carcinoma (HCC) formalin-fixed paraffin-embedded (FFPE) tissues compared to the adjacent noncancerous hepatic tissues. In the current study, we have explored the *in vitro* effect of miR-146a on the malignant phenotypes of HCC cells. MiR-146a mimic could suppress cell growth and increase cellular apoptosis in HCC cell lines HepG2, HepB3, and SNU449, as assessed by spectrophotometry, fluorimetry, and fluorescence microscopy, respectively. Furthermore, western blot showed that miR-146a mimic downregulated EGFR, ERK1/2, and stat5 signalings. These effects were less potent compared to that of a siRNA targeting EGFR, a known target gene of miR-146a. Moreover, miR-146a mimic could enhance the cell growth inhibition and apoptosis induction impact of various EGFR targeting agents. The most potent combination was miR-146a mimic with cetuximab, presenting a synergistic effect. In conclusion, miR-146a plays a vital role in the cell growth and apoptosis of HCC cells and inducing miR-146a level might be a critical targeted molecular therapy strategy for HCC.

## 1. Introduction

Hepatocellular carcinoma (HCC) represents a major form of primary liver malignancy in adults worldwide. The tumorigenesis and development of HCC is typical of a multistage process. Major risk factors for HCC include infection with HBV or HCV, alcoholic liver disease, and most probably nonalcoholic fatty liver disease [1–7]. The progression is considered to deregulate genes that are critical to biological cellular procedures such as cell cycle control, apoptosis, cell migration, and metastasis [8–15]. However, the sensitivity and specificity of these markers remain imperfect [15]. Therefore, new biomarkers are needed to help to understand the causes of hepatocarcinogenesis and to predict response possibilities towards different therapeutic methods.

The current therapies for HCC are challenged. New molecular therapies for HCC include epidermal growth factor receptor (EGFR) inhibitors, for instance, gefitinib, erlotinib [16, 17], cetuximab [18–21], and antiangiogenic compounds, such as bevacizumab [22, 23] and sunitinib [24]. In a phase III trial, patients with advanced HCC treated with the molecular targeted agent sorafenib had an increase in survival of approximately 3 months [25–29]. Nevertheless, new agents must be developed to treat advanced HCC.

MicroRNAs (miRs), small noncoding single-stranded RNAs of 19–24 nucleotides in length, negatively regulate the expression of numerous target genes at the posttranscriptional and/or translational levels and play a vital role in the initiation and deterioration of HCC. Extensive profiling studies over the past several years have indicated that many miRNAs are abnormally expressed in HCC tissues [30–34]. Previously, we found that miR-146a expression was lower in HCC than that in their paracancerous liver tissues. The lower miR-146a expression was also related to advanced clinical TNM stage and distant metastasis [35]. In order to investigate the role of miR-146a on the malignant phenotypes of HCC cells, we performed* in vitro* experiments in the current study to explore the effect of miR-146a on the cell proliferation, viability, caspase-3/7 activity, apoptosis, and regulation of cellular signals in HCC cells lines Hep3B, HepG2, and SNU449. Furthermore, we studied the potential of miR-146a mimic as a therapeutical strategy by combining it and different EGFR target agents, including tyrosine kinase inhibitors (TKIs) gefitinib, erlotinib, and monoclonal antibody cetuximab.

## 2. Materials and Methods

### 2.1. RT-qPCR

The total RNA including miRNA was isolated from HCC cells as reported [36–41]. Combination of RUN6B and let-7a was used as the housekeeping gene for the detection of miR-146a expression. GAPDH was used as internal reference for EGFR mRNA. The primers for miR-146a, RNU6B, and let-7a were included in TaqMan MicroRNA Assays (4427975, Applied Biosystems, Life Technologies Grand Island, NY, USA). Sequence of miRNA and references used in the paper were miR-146a (Applied Biosystems Cat. number 4427975-000468): UGAGAACUGAAUUCCAUGGGUU; RNU6B (Applied Biosystems Cat. number 4427975-001093): CGCAAGGAUGA CACGCAAAUUCGUGAAGCGUUCCAUAUUUUU; let-7a (Applied Biosystems Cat. number 4427975-000377): UGAGGUAGUAGGUUGUAUAGUU. The reverse primers were also used for the reverse transcription with TaqMan MicroRNA Reverse Transcription Kit (4366596, Applied Biosystems, Life Technologies Grand Island, NY, USA) in a total volume of 10 *μ*L. Real-time qPCR for miRNA was performed with Applied Biosystems PCR7900. The alteration ratio of miR-146a was (1–1/2^ΔΔCq^) × 100% [37, 41]. Real time RT-qPCR for EGFR was performed as reported [36, 38–41].

### 2.2. Inhibition and Reexpression of mir-146a in HCC Cells

The human HCC-derived cell lines HepG2 (HB-8065), HepB3 (HB-8064), and SNU449 (CRL-2234) were purchased from the American Type Culture Collection (ATCC, Rockville, MD, USA). They were cultured as previously described [42–44]. All* in vitro* experiments were performed in triplicate. HCC cells were planted in 24-well plates (2.5 × 10^4^ cells per well) or 96-well plates (2.5 × 10^3^ cells per well) and incubated at 37°C for 24 h. The cells were transfected with miR-146a inhibitor, miRNA inhibitor negative control, miR-146a mimic, and miRNA mimic negative control, respectively (Ambion, Life Technologies Grand Island, NY, USA) at a final concentration of 200 nmol/L for 96 h using combiMAGnetofection (OZ BIOSCIENCES, Marseille Cedex 9, France) in accordance with manufacturer's procedure. MiRNA mimics are small, chemically modified double-stranded RNA molecules designed to specifically bind to and mimic endogenous miRNA molecules. They can enable miRNA functional analysis by upregulation of miRNA activity. MiRVana miRNA Mimic Negative Control Number 1 was used as miRNA mimic negative control in the current study and it is a random sequence miRNA mimic molecule that has been extensively tested in human cell lines and tissues and validated not to produce identifiable effects on known miRNA function. MiRNA inhibitors are chemically modified, single-stranded nucleic acids designed to specifically bind to and inhibit endogenous miRNA molecules. These ready-to-use inhibitors can be introduced into cells using transfection or electroporation parameters similar to those used for siRNAs. Anti-miR Negative Control Number 1 was used as inhibitor negative control in the present study and it is a random sequence anti-miR molecule that has been extensively tested in human cell lines and tissues and validated to produce no identifiable effects on known miRNA function. EGFR TKIs gefitinib, erlotinib, and EGFR-specific monoclonal antibody cetuximab (2 mg/mL, 13.719 *μ*M) were purchased from Selleckchem, Munich, Germany. All agents were prepared as described previously [36–41]. The EGFR specific siRNA was described previously [36–39] (sequence: GCAAAGTGTGTAACGGAATAGGTAT). The EGFR specific siRNA was transfected into HCC cells with the same method as above.

### 2.3. Cell Biological Function Detections

Cell proliferation, cell viability, apoptosis and nuclear morphology, and caspase-3/7 activity were performed as described previously [36–41, 43, 44] to study the effects of miR-146a inhibitor and miR-146a mimic. Western blot was performed as described previously [36–41, 43, 44]. The following primary antibodies were used: phospho-EGFR (Tyr1173, clone 9H2, Upstate), total-EGFR (2232, Cell Signaling Technology), phospho-AKT/PKB (pS473, Invitrogen), total-AKT (9272, Cell Signaling Technology), phospho-ERK1/2 (pTpY185/187, Invitrogen), total-ERK1/2 (9102, Cell Signaling Technology), phospho-stat5 (pY694, BD Biosciences), total-stat5 (9363, Cell Signaling Technology), caspase-3 (8G10, 9665, Cell Signaling Technology), and *β*-actin (Sigma-Aldrich N.V.).

### 2.4. Statistical Analysis

SPSS19.0 (Munich, Germany) was performed for statistical analysis. Results were representative of minimal three independent experiments unless stated otherwise. Values were presented as the mean ± standard deviation (SD). One-way analysis of variance (ANOVA) test was used to analyze significance between different groups. The least significant difference (LSD) method of multiple comparisons between two groups was applied when the probability for ANOVA was statistically significant. Statistical significance was determined at a *P* < 0.05 level. In the analysis of additivity and synergism, the theoretical zero-interaction (exactly additive) dose-response curve for each miR-146a mimic + other agent combination was calculated by applying Bliss independence criterion [45] and was also assessed by the Biosoft CalcuSyn program (Ferguson, MO, USA). The combination index (CI) was used to express synergism (CI < 1), additive effect (CI = 1), or antagonism (CI > 1) [37, 38, 40, 41, 43, 46].

## 3. Results

### 3.1. MiR-146a Inhibited Cell Growth in HCC Cells

**Figure 1:**
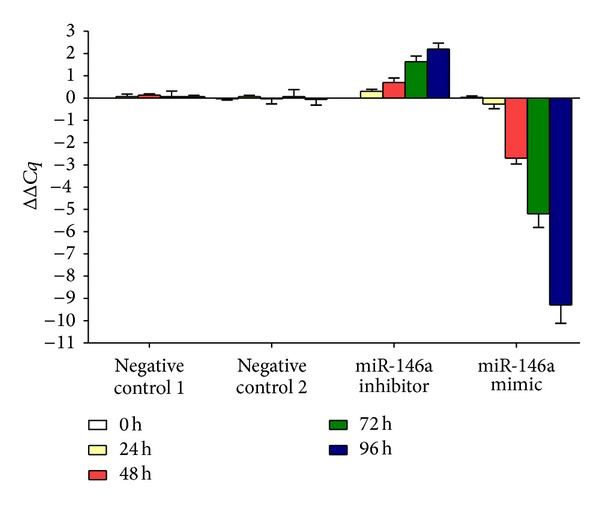
Transfection efficiency of miR-146a inhibitor and miR-146a mimic in HepG2 cells. HepG2 cells (2.5 × 10^4^ cells per well in 24-well plate) were planted and transfected with miR-146a inhibitor, miR-146a mimic, and their negative controls up to 96 h. Real time RT-qPCR was performed to detect the level of miR-146a and delta delta Cq was calculated. Negative control 1: negative control of miRNA inhibitor; Negative control 2: negative control of miRNA mimic.

**Figure 2:**
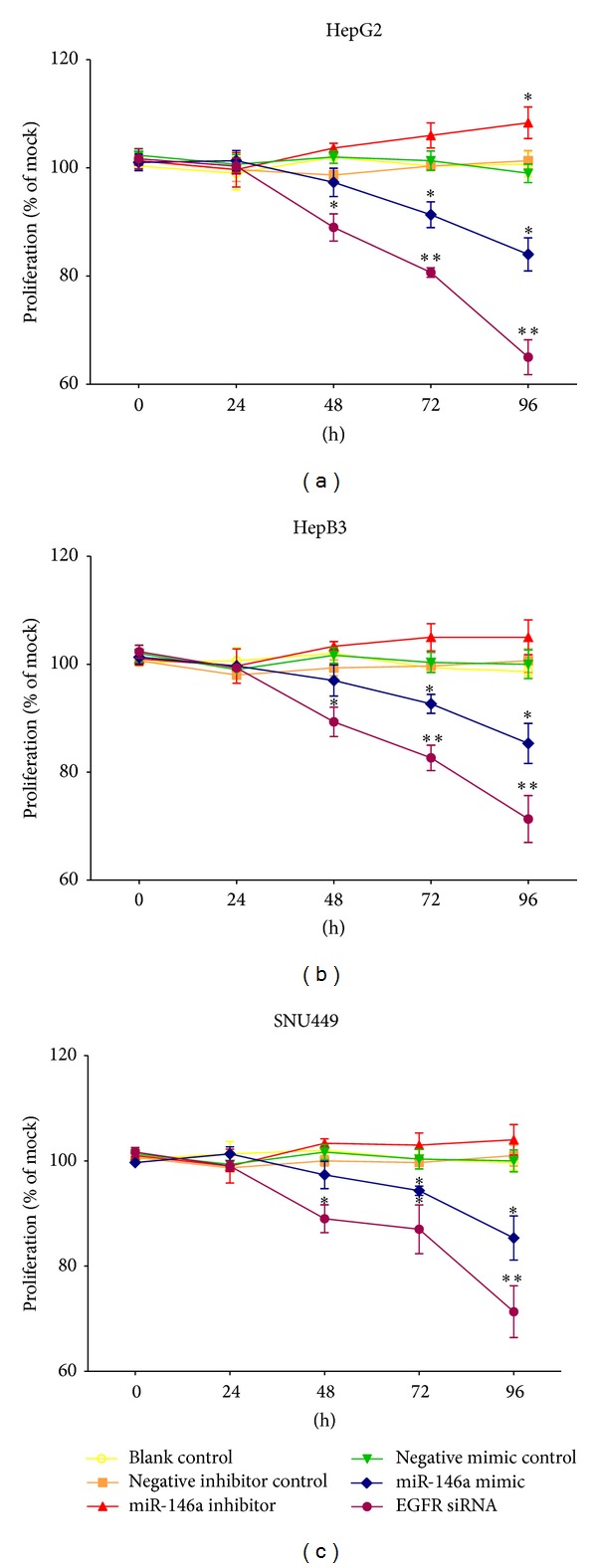
Time dependent effect of miR-146a on cell proliferation in HCC cell lines. HepG2, HepB3, and SNU449 cells (2.5 × 10^3^ cells per well in 96-well plate) were cultured for 24 h and then transfected with miR-146a inhibitor, miR-146a mimic, EGFR siRNA, and their negative controls (200 nM) up to another 96 h. Cell proliferation was assessed per day with MTS assay (CellTiter 96 Aqueous One Solution Cell Proliferation Assay). *P < 0.05, **P < 0.01, compared to blank and negative controls at the same time point.

**Figure 3:**
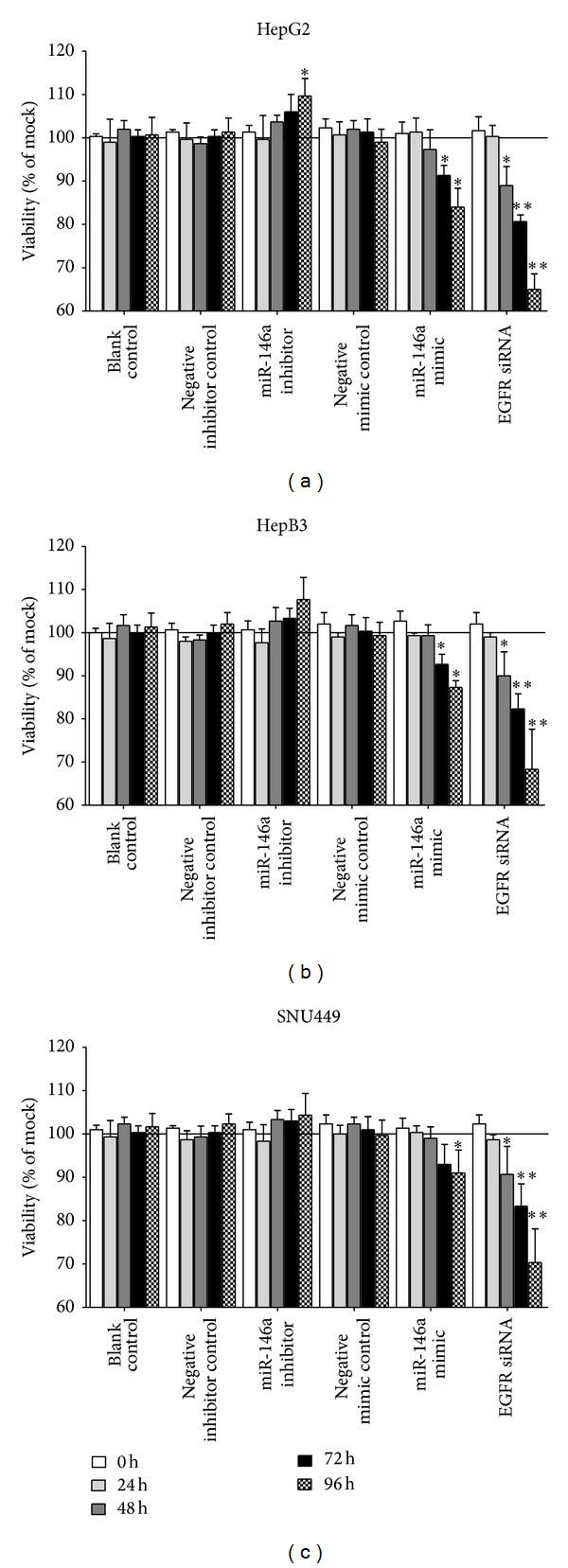
Time dependent effect of miR-146a on cell viability in HCC cell lines. HepG2, HepB3, and SNU449 cells (2.5 × 10^3^ cells per well in 96-well plate) were cultured for 24 h and then transfected with miR-146a inhibitor, miR-146a mimic, EGFR siRNA, and their negative controls (200 nM) up to another 96 h. Cell viability was monitored using fluorimetric detection of resorufin (CellTiter-Blue Cell Viability Assay). *P < 0.05, **P < 0.01, compared to blank and negative controls at the same time point.

**Figure 4:**
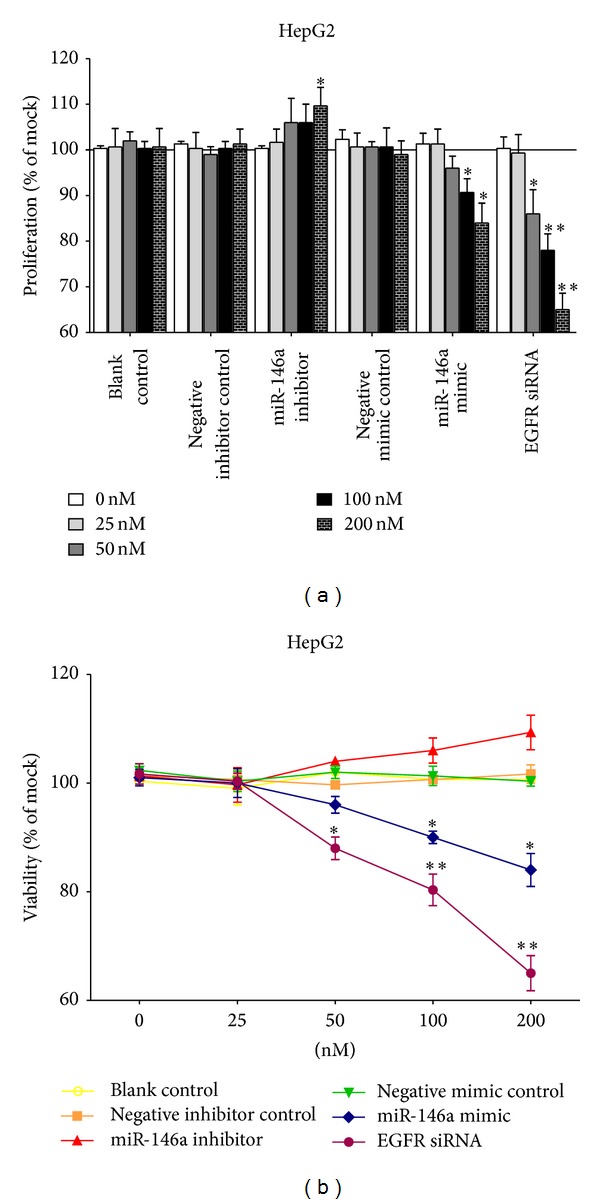
Dose dependent effect of miR-146a on cell growth in HCC cell line HepG2. HepG2 cells (2.5 × 10^3^ cells per well in 96-well plate) were cultured for 24 h and transfected with miR-146a inhibitor, miR-146a mimic, EGFR siRNA, and their negative controls with increasing concentrations for 96 h (0–200 nM). (a) Cell proliferation tested with MTS assay. (b) Cell viability detected using fluorimetric detection of resorufin (CellTiter-Blue Cell Viability Assay). *P < 0.05, **P < 0.01, compared to blank and negative controls with the same concentration.

Transfection efficiency was first confirmed using real time RT-qPCR (Figure 1). The effect of miR-146a on cell growth was detected using three independent assays, including MTS tetrazolium assay, fluorimetric resorufin viability assay, and Hoechst 33342/propidium iodide (PI) double fluorescent chromatin staining, respectively. MTS tetrazolium assay revealed that cell proliferation increased slightly in HepG2 cells 96 h after transfection compared to blank and negative controls with miR-146a inhibitor. MiR-146a inhibitor exerted no influence on HepB3 or SNU449 cells. After transfection with the miR-146a mimic, a large reduction in proliferation was examined at 72 and 96 h in all the three cell lines tested, although less than the effect of siRNA targeting EGFR (Figure 2). The cell growth inhibitory effect showed a time dependent manner. To verify these results, the effect on cell viability was assessed by using a fluorimetric resorufin viability assay (Figure 3) and Hoechst 33342/PI double fluorescent chromatin staining (data not shown), which largely mirrored the results from MTS assay. The effect of miR-146a on cell growth suppression showed also a dose dependent manner (Figure 4).

### 3.2. MiR-146a Mimic Induced Apoptosis in HCC Cells

**Figure 5:**
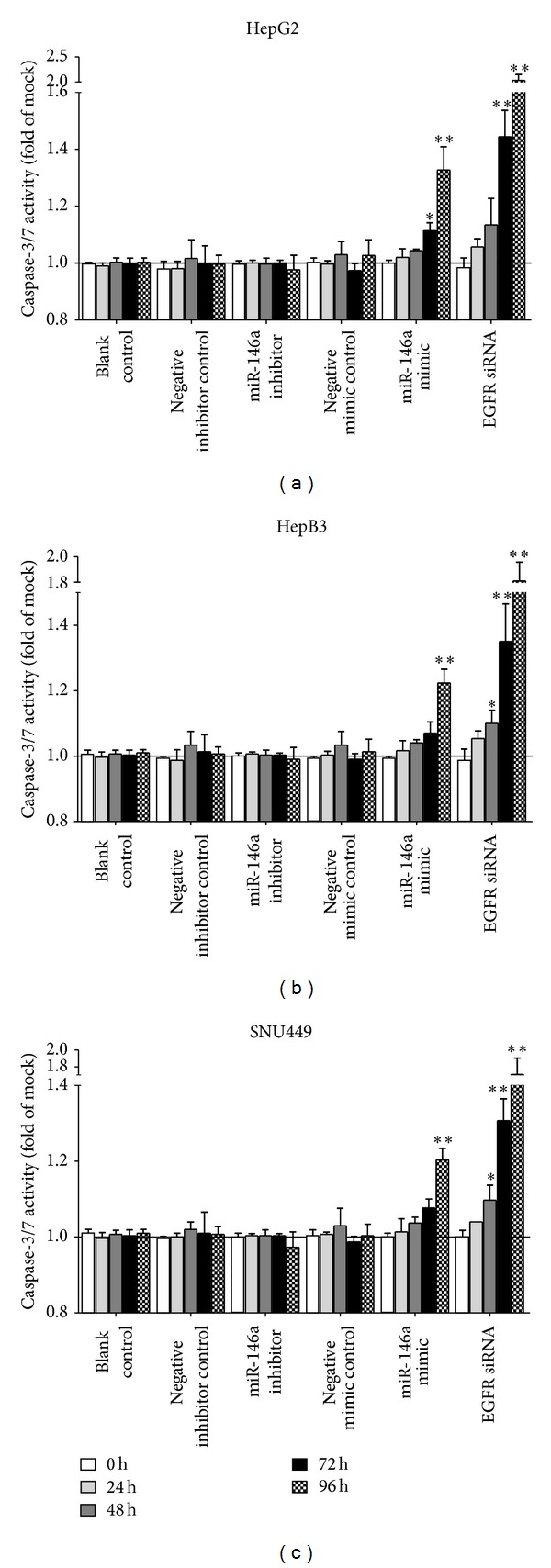
Time dependent effect of miR-146a on cell caspase-3/7 activity in HCC cell lines. HepG2, HepB3, and SNU449 cells (2.5 × 10^3^ cells per well in 96-well plate) were cultured for 24 h and then transfected with miR-146a inhibitor, miR-146a mimic, EGFR siRNA, and their negative controls (200 nM) up to another 96 h. Caspase-3/7 activity was detected using Apo-ONE Homogeneous Caspase-3/7 Assay. *P < 0.05, **P < 0.01, compared to blank and negative controls at the same time point.

**Figure 6:**
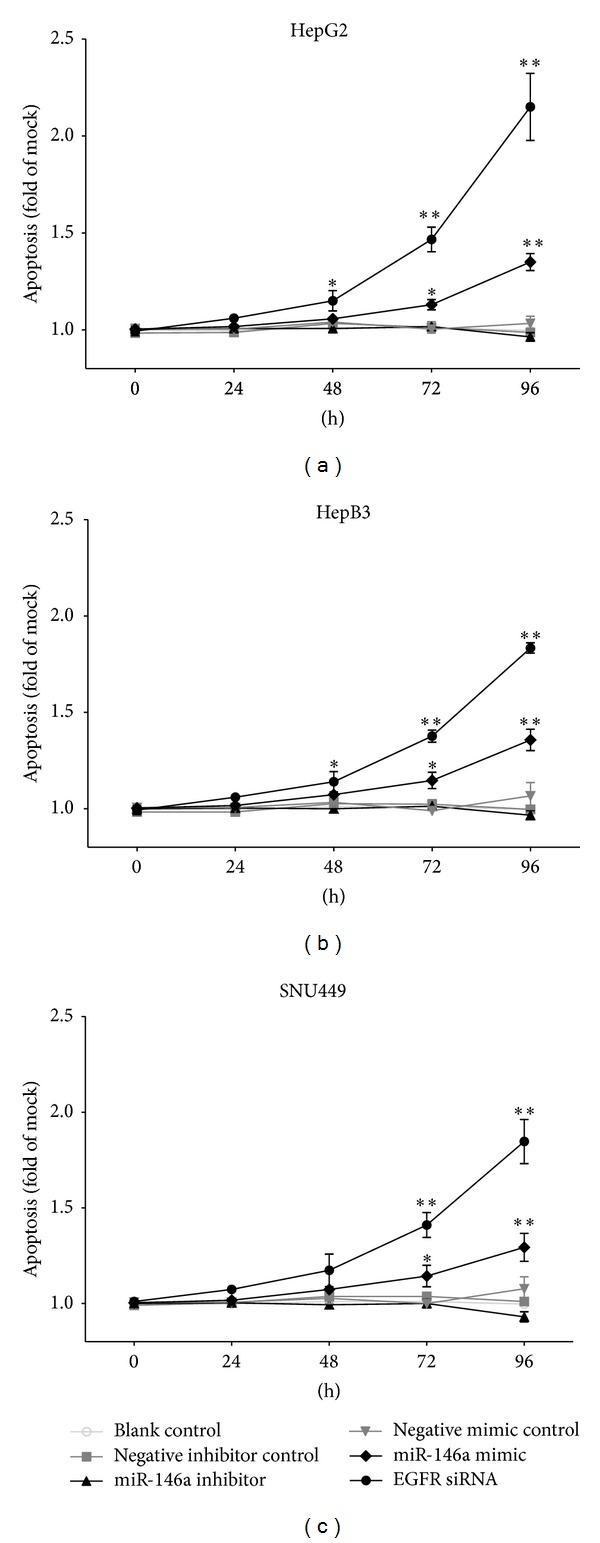
Time dependent effect of miR-146a on apoptosis in HCC cell lines. HepG2, HepB3, and SNU449 cells (2.5 × 10^3^ cells per well in 96-well plate) were cultured for 24 h and then transfected with miR-146a inhibitor, miR-146a mimic, EGFR siRNA, and their negative controls (200 nM) up to another 96 h. Hoechst 33342/propidium iodide (PI) double fluorescent chromatin staining was used to analyze apoptosis. *P < 0.05, **P < 0.01, compared to blank and negative controls at the same time point.

**Figure 7:**
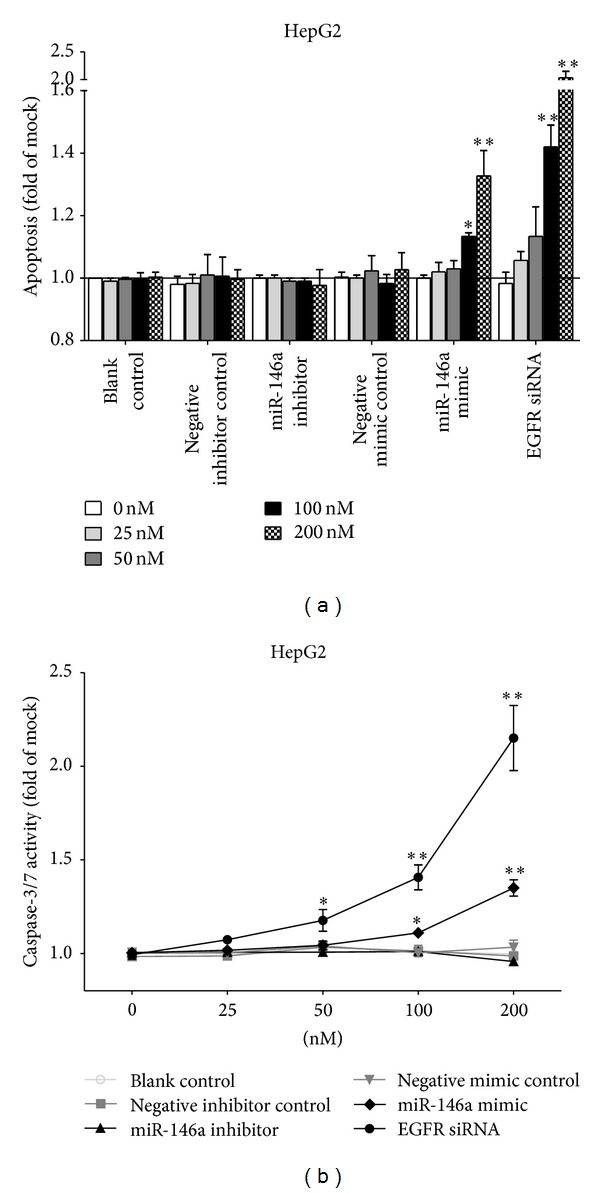
Dose dependent effect of miR-146a on cell caspase-3/7 activity and apoptosis in HCC cell line HepG2. HepG2 cells (2.5 × 10^3^ cells per well in 96-well plate) were cultured for 24 h then transfected with miR-146a inhibitor, miR-146a mimic, EGFR siRNA, and their negative controls (0–200 nM) up to another 96 h. (a) Cellular apoptosis tested with Hoechst 33342/propidium iodide (PI) double fluorescent chromatin staining. (b) Cell caspase-3/7 activity detected using Apo-ONE Homogeneous Caspase-3/7 Assay. *P < 0.05, **P < 0.01, compared to blank and negative controls with the same concentration.

**Figure 8:**
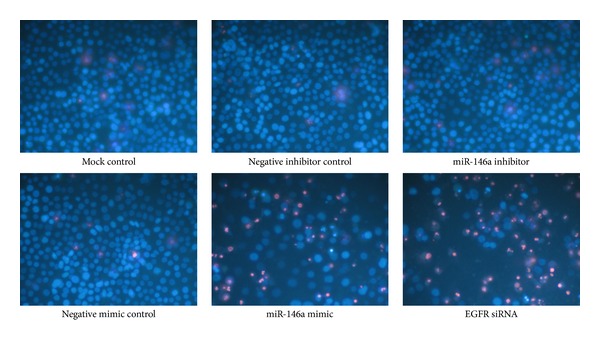
MiR-146a mimic suppressed cell growth and induced apoptosis with Hoechst 33342/propidium iodide (PI) double fluorescent chromatin staining. HepG2 cells (2.5 × 10^3^ cells per well in 96-well plate) were cultured for 24 h then transfected with miR-146a inhibitor, miR-146a mimic, EGFR siRNA, and their negative controls (200 nM) up to another 96 h. The effect on apoptosis was assessed and compared to mock and negative controls, ×200.

To validate whether miR-146a is able to influence apoptosis, the CellTiter-Blue assay was multiplexed with a fluorescent caspase-3/7 assay. The results showed that with the miR-146a inhibitor, caspase-3/7 activity was slightly less than the blank and negative controls, but indicated no significant change. However, with the miR-146a mimic, caspase-3/7 activity was markedly enhanced in all three HCC cell lines tested (Figure 5) with a time and dose dependent manner. Similar to the result of cell growth, the effect of miR-146a on caspase activity was much milder than that of siRNA targeting EGFR. The time and dose dependent effect on apoptosis was confirmed microscopically by Hoechst 33342 and PI double fluorescent staining (Figures 6, 7 and 8).

### 3.3. Contribution of miR-146a in Relevant Cellular Signaling

**Figure 9:**
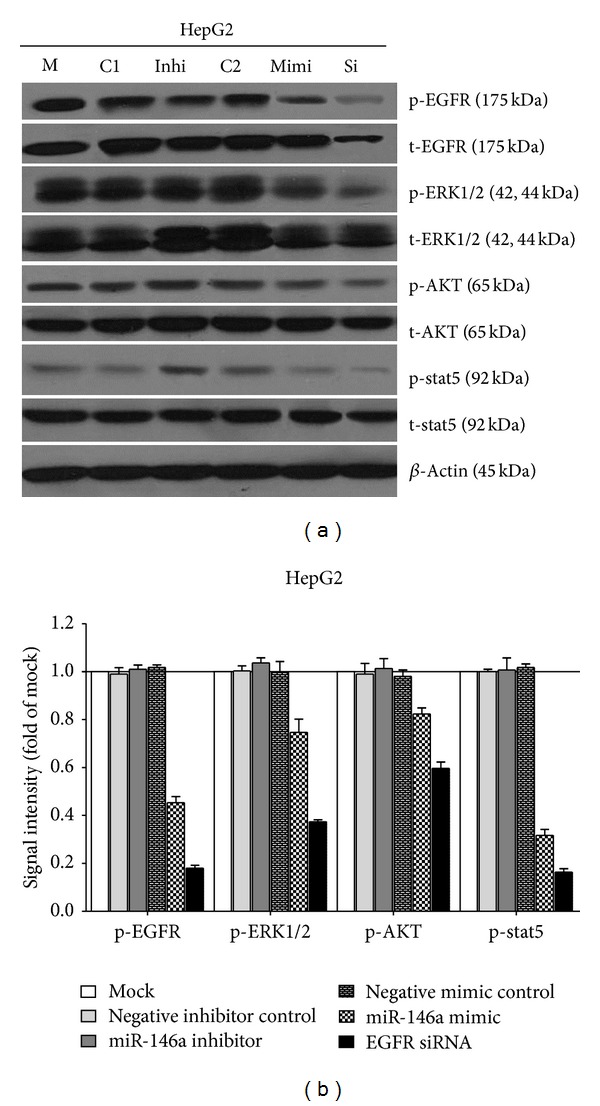
Effect of miR-146a on EGFR and its downstream pathway signals in HCC HepG2 cells. HepG2 cells (2.5 × 10^4^ cells per well in 24-well plate) were cultured for 24 h then transfected with miR-146a inhibitor, miR-146a mimic, EGFR siRNA, and their negative controls (200 nM) up to another 96 h. Western blot and signal intensity of the bands were shown. Antibodies included phospho-EGFR (p-EGFR), total-EGFR (t-EGFR), p-ERK1/2, t-ERK1/2, p-AKT, t-AKT, p-stat5, t-stat5, and *β*-Actin. M: mock control; C1: negative control for miRNA inhibitor; Inhi: miR-146a inhibitor; C2: negative control for miRNA mimic; Mimi: miR-146a mimic; Si: EGFR siRNA.

To investigate the contribution of miR-146a in the regulation of cellular signaling, we examined the signaling of EGFR, AKT, ERK, and stat pathways by using western blot, which are related to cell survival, apoptosis, and invasion. These pathways were influenced little with miR-146a inhibitor transfection. However, the phospho-EGFR, phospho-ERK1/2, and phospho-stat5 were downregulated by miR-146a mimic 96 h after transfection, although weaker than EGFR siRNA (Figure 9).

### 3.4. Synergistic Effect of miR-146a Mimic and Cetuximab

**Figure 10:**
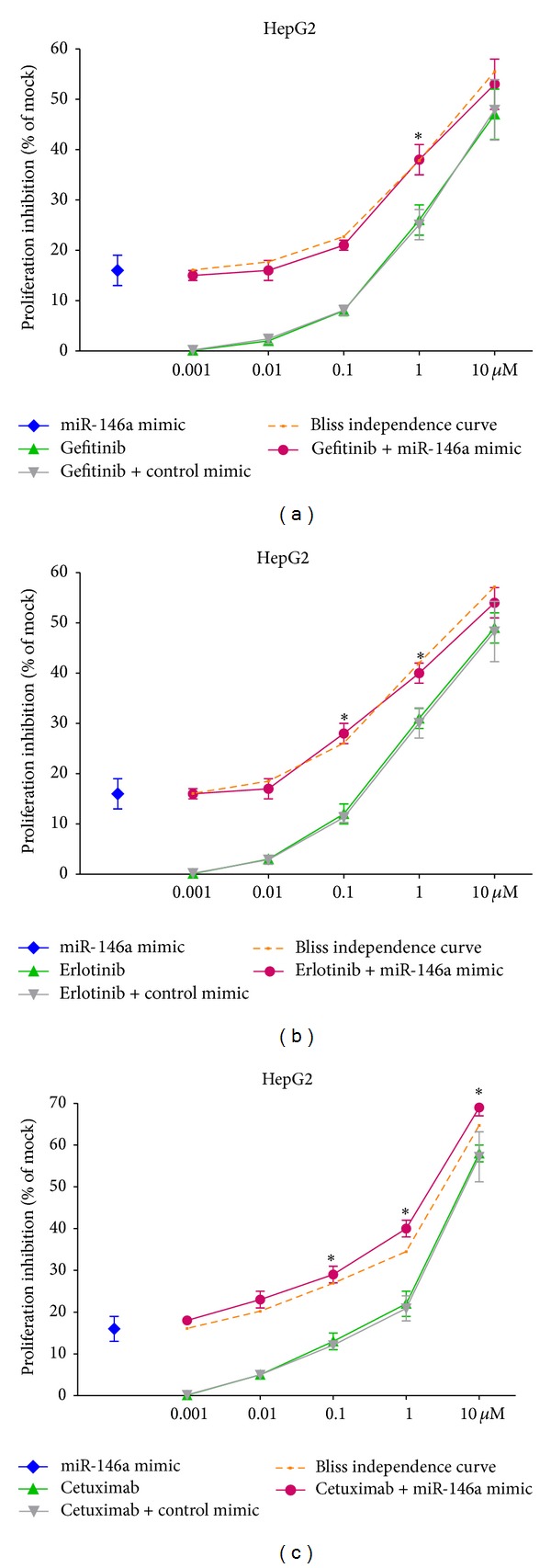
miR-146a mimic enhanced the growth inhibitory effect of EGFR targeting agents in HCC HepG2 cells. HepG2 cells (2.5 × 10^3^ cells per well in 96-well plate) were cultured for 24 h then transfected with miR-146a mimic and negative control (200 nM). Meanwhile, gefitinib, erlotinib, and cetuximab were added and the cells were cultured up to another 96 h. MTS was performed as above and the proliferation inhibition rate was calculated. Bliss independence curve indicated the theoretical situation in which the combinational effect of miR-146a mimic and other agents was exactly additive. *P < 0.05, compared to agent alone. Bliss independence criterion was applied to analyze the theoretical additive effect.

**Figure 11:**
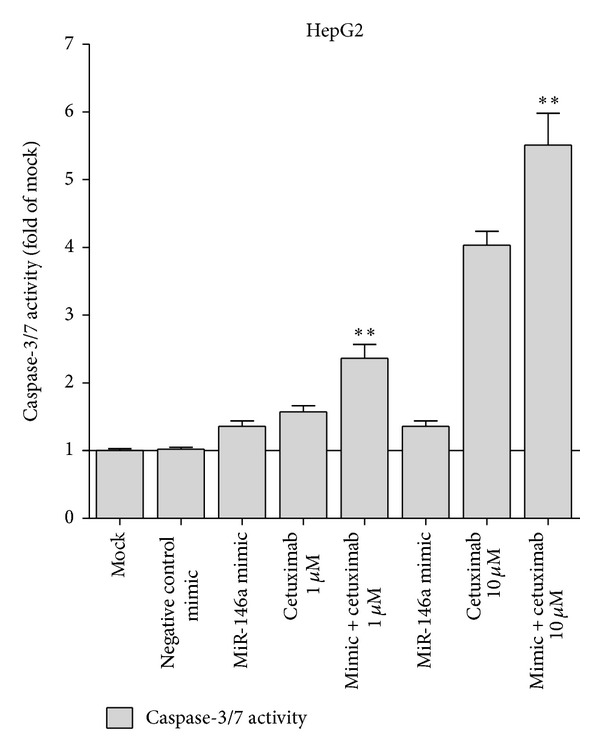
miR-146a mimic increased the caspase-3/7 activity of cetuximab in HCC HepG2 cells. HepG2 cells (2.5 × 10^3^ cells per well in 96-well plate) were cultured for 24 h then transfected with miR-146a mimic and negative control (200 nM). Meanwhile, cetuximab was added and the cells were cultured up to another 96 h. Caspase activity was performed as above. **P < 0.01, compared to agent alone of the same concentration.

**Figure 12:**
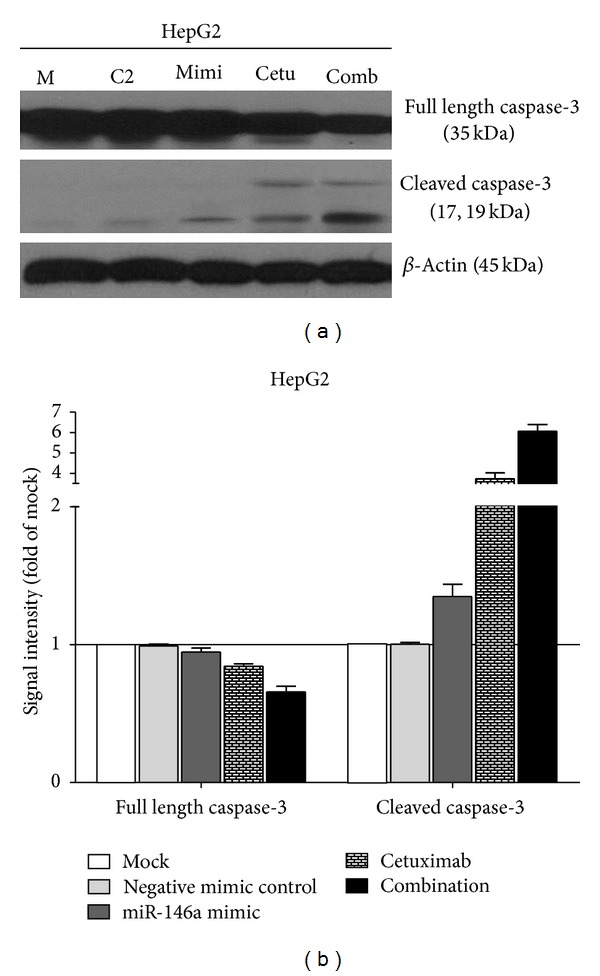
Combination of miR-146a mimic and cetuximab induced more cleaved caspase-3 in HCC HepG2 cells. HepG2 cells (2.5 × 10^4^ cells per well in 24-well plate) were cultured for 24 h then transfected with miR-146a mimic and negative control (200 nM). Meanwhile, cetuximab was added and the cells were cultured up to another 96 h. Western blot and signal intensity of the bands were shown. Antibodies include full length caspase-3, cleaved caspase-3, and *β*-Actin. M: mock control; C2: negative control for miRNA mimic; Mimi: miR-146a mimic; Cetu: cetuximab; comb: combination of miR-146a and cetuximab.

**Figure 13:**
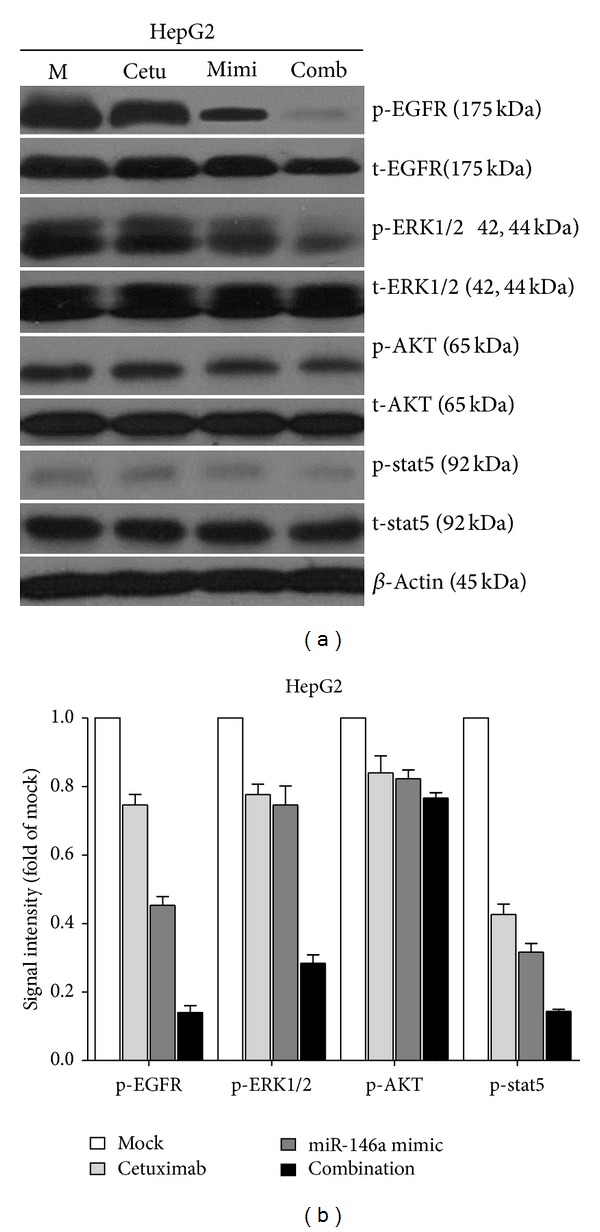
Effect of combining miR-146a mimic and cetuximab on EGFR and its downstream pathway signals in HCC HepG2 cells. HepG2 cells (2.5 × 10^4^ cells per well in 24-well plate) were cultured for 24 h then transfected with miR-146a mimic and negative control (200 nM). Meanwhile, cetuximab was added and the cells were cultured up to another 96 h. Western blot and signal intensity of the bands were shown. Antibodies included phospho-EGFR (p-EGFR), total-EGFR (t-EGFR), p-ERK1/2, t-ERK1/2, p-AKT, t-AKT, p-stat5, t-stat5, and *β*-Actin. M: mock control; Cetu: cetuximab; Mimi: miR-146a mimic; comb: combination of cetuximab and miR-146a mimic.

**Figure 14:**
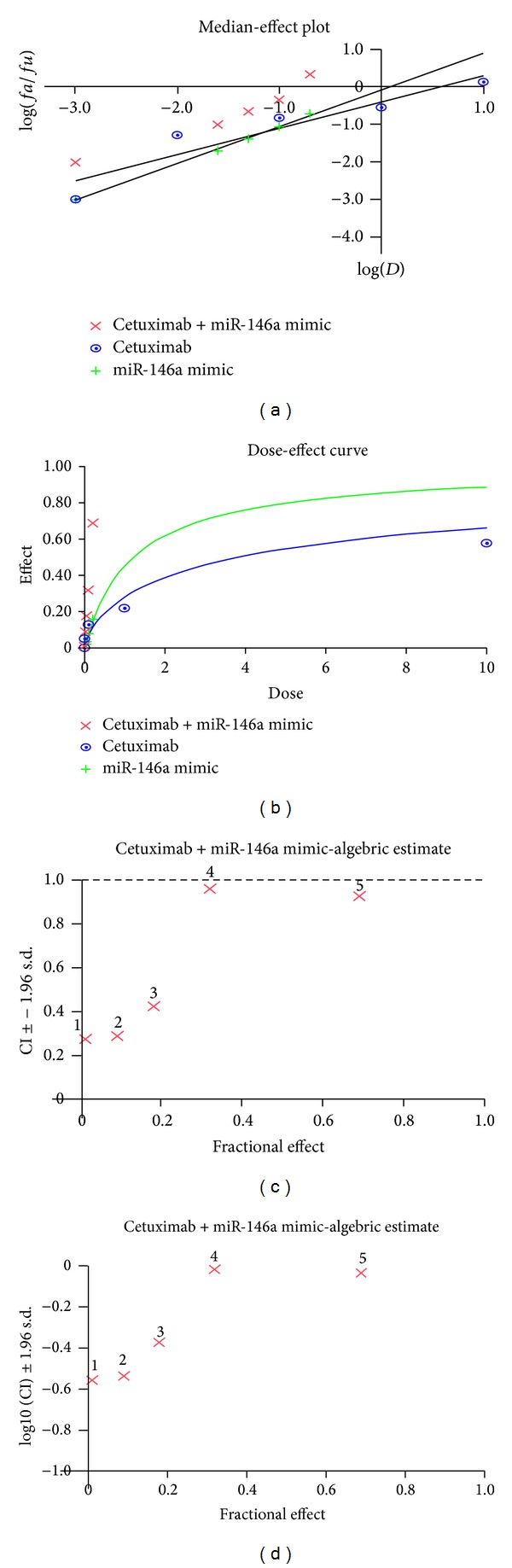
Combinational effect of cetuximab and miR-146a mimic in HepG2 cells. Cetuximab (0.001, 0.01, 0.1, 1, and 10 *μ*M) was combined with miR-146a mimic (0.001, 0.025, 0.05, 0.1, and 0.2 *μ*M) and cell proliferation was detected by MTS assay. Biosoft CalcuSyn program was used to calculate (a) median-effect plot, (b) dose-effect curve, and (c) CI. This indicates synergistic effect since CI < 1, (d) log 10 (CI).

EGFR is a confirmed target gene of miR-146a as previously reported [41, 47, 48]. We desired to explore the combinatorial effect of miR-146a mimic and agents targeting EGFR (small molecular inhibitors or monoclonal antibody), using the colorimetric MTS formazan proliferation assay and caspase activity assay. The inhibition of cell proliferation was much stronger with miR-146a mimic in combination with gefitinib, erlotinib, or cetuximab, compared to single drug or single miR-146a mimic in HepG2 cells (Figure 10). However, for gefitinib and erlotinib, the proliferation curve of the combinatorial treatment was not significantly higher than the Bliss independence curve, which indicated an additive effect. Surprisingly, the combination of miR-146a mimic and cetuximab showed an extremely higher inhibitory effect compared to the Bliss independence curve, which suggested a possible synergy in cell growth suppression (Figure 10). Furthermore, the combination of miR-146a mimic and cetuximab also gave a significantly higher caspase-3/7 signal than single treatment (Figure 11). In addition, western blot proved that with the combination of miR-146a mimic and cetuximab, the caspase-3 protein level was reduced and cleaved caspase-3 expression was increased (Figure 12). Meanwhile, the EGFR and downstream pathway signals were also further downregulated with miR-146a mimic combined with cetuximab, especially p-EGFR and p-ERK1/2 (Figure 13). To verify the additive or synergistic nature of combining EGFR targeted agents with the miR-146a mimic, a CI was calculated [37, 38, 40, 41, 45]. This unambiguously showed that the effect of gefitinib or erlotinib and miR-146a was additive (data not shown). With regard to the combination of cetuximab and miR-146a mimic, the effect was indeed entirely synergistic (Figure 14).

## 4. Discussion

Previously, we found that the relevant miR-146a expression in the HCC tissues and cultured cells was significantly lower than that in the adjacent noncancerous hepatic tissues. Moreover, miR-146a expression in early clinical stages (I and II) was remarkably higher than that in advanced stages (III and IV). Lower level of miR-146a was also found in HCC patients with metastasis compared with those without metastasis [35]. These results strongly suggest that miR-146a acts as a tumor suppressor miRNA in HCC. To further explore the function of miR-146a on the malignant phenotypes of HCC cell, we performed a series of* in vitro* experiments.

We first investigated the contribution of miR-146a to cell growth and apoptosis in HCC cells and also compared the effect of miR-146a mimic to the siRNA specially targeting EGFR mRNA, since EGFR was documented as a target of miR-146a in various cancers [41, 47–50]. In the current study, the EGFR protein was downregulated in HCC HepG2 cells after transfection of miR-146a mimic, which implies that EGFR is a target of miR-146a in HCC. The miR-146a mimic decelerated the cell growth in all the cell lines tested (HepG2, HepB3, and SNU449), using three independent assays. Additionally, miR-146a mimic enhanced the caspase-3/7 activity and induced apoptosis in HCC cell lines. This could partially be explained by the downregulation of EGFR and the downstream pathways as shown by western blot, especially ERK1/2 and stat5, though HCC HepG2 expresses low level of p-stat5 at the basic line. However, the effect of miR-146a mimic on inhibiting cell growth and inducing apoptosis was much weaker than that of an EGFR-specific-siRNA. Since different mechanism could be involved in the process of EGFR gene silencing, probably much higher dosage of miR-146a mimic is needed to reach the same effect of EGFR siRNA. The miR-146a inhibitor had little effect on the function of HCC cells, which could be related to the limited downregulation of miR-146a level. With miR-146a inhibitor, the ΔΔCq was about 2 times higher than the negative controls, in contrast with miR-146a mimic; the ΔΔCq was about 9 times lower than the controls.

The suppressive role of miR-146a on the malignant phenotype of HCC cells makes it tempting to develop novel therapeutical strategies of inducing miR-146a levels. When we combined the miR-146a mimic together with different EGFR TKIs or cetuximab in HCC cells, miR-146a mimic was found to enhance the cell proliferation inhibition and apoptosis induction by TKIs and cetuximab, the strongest effect obtained when combined with cetuximab. Synergistic effect was obtained with the combination of miR-146a mimic and cetuximab. Western blot also confirmed that the EGFR, ERK1/2, and stat5 pathways were strongly inhibited with this combination. Since EGFR siRNA has stronger effect than miR-146a mimic, we wondered if the combination of EGFR siRNA and cetuximab could produce even more potent synergistic effect. Indeed, EGFR siRNA plus cetuximab have very powerful effect on cell growth inhibition and apoptosis induction (unpublished data on file). The level of miR-146a in cancer cells can also be upregulated by other agents. For example, Li et al. found that reexpression of miR-146a by nontoxic “natural agents,” including 3,3′-diindolylmethane, and isoflavone, has antitumor effects in pancreatic cancer [47].

## 5. Conclusions

Since miR-146a was found to be related to the HCC metastases, further* in vitro* studies are planned to identify the mechanism of miR-146a in metastasis. Other possible target genes of miR-146a, such as TRAF6, IRAK1, and NUMB, will also be tested. On the other hand, cell growth inhibition and apoptosis induction by miR-146a mimic represent great relevance due to its possible therapeutic potential. The application of miR-146a mimic might thus be a promising approach to HCC therapies in the future, for both* in vivo* studies and clinic trials.
