# Corrigendum to “Synergistic Effect of MiR-146a Mimic and Cetuximab on Hepatocellular Carcinoma Cells”

**DOI:** 10.1155/2020/9375214

**Published:** 2020-12-13

**Authors:** Suning Huang, Rongquan He, Minhua Rong, Yiwu Dang, Gang Chen

**Affiliations:** ^1^Department of Radiotherapy, First Affiliated Hospital of Guangxi Medical University, 6 Shuangyong Road, Nanning, Guangxi Zhuang Autonomous Region 530021, China; ^2^Department of Medical Oncology, First Affiliated Hospital of Guangxi Medical University, 6 Shuangyong Road, Nanning, Guangxi Zhuang Autonomous Region 530021, China; ^3^Research Department, Affiliated Cancer Hospital, Guangxi Medical University, 71 Hedi Road, Nanning, Guangxi Zhuang Autonomous Region 530021, China; ^4^Department of Pathology, First Affiliated Hospital of Guangxi Medical University, 6 Shuangyong Road, Nanning, Guangxi Zhuang Autonomous Region 530021, China

In the article titled, “Synergistic Effect of MiR-146a Mimic and Cetuximab on Hepatocellular Carcinoma Cells” [1], the authors reported that they had misspelt the name of a cell line and supplied the incorrect figure for publication. The correct name of the cell line should be Hep3B, instead of HepB3. The correct Figure 8 is displayed below. The results and conclusions described therein are not affected by these corrections.

## Figures and Tables

**Figure 1:**
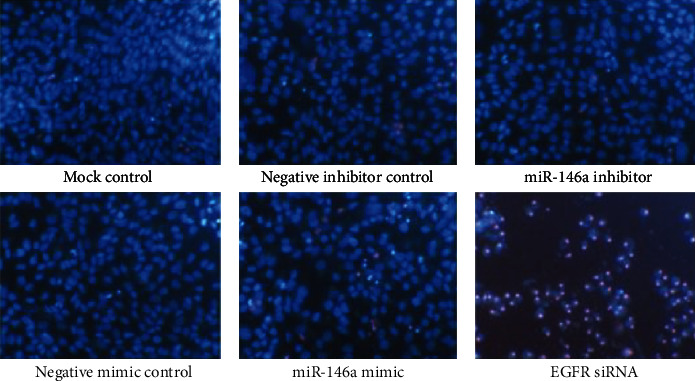
MiR-146a mimic induced apoptosis with Hoechst 33342/propidium iodide (PI) double fluorescent chromatin staining. HepG2 cells (2.5 × 10^3^ cells per well in a 96-well plate) were cultured for 24 h then transfected with miR-146a inhibitor, miR-146a mimic, EGFR siRNA, and their negative controls (200 nM) up to another 96 h. The effect on apoptosis was assessed and compared to mock and negative controls, ×200.
